# IMP2/p62 induces genomic instability and an aggressive hepatocellular carcinoma phenotype

**DOI:** 10.1038/cddis.2015.241

**Published:** 2015-10-01

**Authors:** S M Kessler, S Laggai, A Barghash, C S Schultheiss, E Lederer, M Artl, V Helms, J Haybaeck, A K Kiemer

**Affiliations:** 1 Department of Pharmacy, Pharmaceutical Biology, Saarland University, Saarbruecken, Germany; 2 Institute of Pathology, Medical University of Graz, Graz, Austria; 3 Center for Bioinformatics, Saarland University, Saarbruecken, Germany; 4 Saarbruecken Graduate School of Computer Science, Saarbruecken, Germany; 5 Institute of Human Genetics, Medical University of Graz, Graz, Austria

## Abstract

Hepatocellular carcinoma (HCC) represents the third leading cause of cancer-related deaths and commonly develops in inflammatory environments. The *IGF2* mRNA-binding protein *IMP2-2/IGF2BP2-2/p62* was originally identified as an autoantigen in HCC. Aim of this study was to investigate a potential pathophysiological role of p62 in hepatocarcinogenesis. Human HCC tissue showed overexpression of *IMP2*, which strongly correlated with the fetal markers *AFP* and *DLK1*/*Pref-1*/*FA-1* and was particularly elevated in tumors with stem-like features and hypervascularization. Molecular classification of IMP2-overexpressing tumors revealed an aggressive phenotype. Livers of mice overexpressing the IMP2 splice variant p62 highly expressed the stem cell marker DLK1 and secreted DLK1 into the blood. p62 was oncogenic: diethylnitrosamine (DEN)-treated *p62* transgenic mice exhibited a higher tumor incidence and multiplicity than wild types. Tumors of transgenics showed a more aggressive and stem-like phenotype and displayed more oncogenic chromosomal aberrations determined with aCGH analysis. DEN-treated *p62* transgenic mice exhibited distinct signs of inflammation, such as inflammatory cytokine expression and oxidative stress markers, that is, thiobarbituric acid-reactive substance (TBARS) levels. Reactive oxygen species (ROS) production was elevated in HepG2 cells, which either overexpressed p62 or were treated with DLK1. p62 induced this ROS production by a DLK1-dependent induction and activation of the small Rho-GTPase RAC1, activating NADPH oxidase and being overexpressed in human HCC. Our data indicate that p62/IMP2 promotes hepatocarcinogenesis by an amplification of inflammation.

Hepatocellular carcinoma (HCC) is the third leading cause of cancer-related death.^1^ In most cases, HCC develops based on an inflammatory etiology, namely chronic hepatitis provoked by either viruses, or alcoholic, and non-alcoholic steatohepatitis. Elevated reactive oxygen species (ROS) generation represents a hallmark of inflammation and promotes carcinogenesis.^2^

The insulin-like growth factor 2 (*IGF2*) mRNA-binding protein p62/IMP2-2/IGF2BP2-2 represents a shortened splice variant of *IMP2*, but harboring the identical mRNA-binding domain.^3^ Although p62 was originally identified as an autoantigen in an HCC patient,^4^ a functional impact of p62 or IMP2 on hepatocarcinogenesis has not been described in detail as yet. Still, other members of the IMP family, that is, IMP1 and IMP3, were reported to promote HCC^5, 6^ and other tumors.^7, 8^

*p62* transgenic mice expressing the transgene exclusively in the liver develop steatosis^9, 10^ and are more prone to develop steatohepatitis.^11^ The animals express elevated levels of the imprinted genes *H19* and *Igf2*,^10^ suggesting an effect of p62 on a specific cluster of imprinted genes.^12^ IGF2 displays a key regulator in mammalian growth through metabolic and growth-promoting effects. Whereas p62 was recently reported to exert its lipogenic actions via IGF2,^9^ its anti-apoptotic actions are independent of IGF2.^13^ In addition, IMP2 was suggested to promote HCC cell survival.^14^

Employing transgenic animals and hepatoma cells we here show that p62 induces an aggressive HCC phenotype, which is linked to inflammatory and oxidant actions of p62. Analyses of publicly available human HCC gene expression data further support p62 as a marker of human HCC with poor prognosis.

## Results

### p62 expression correlates with DLK1 and promotes hepatocarcinogenesis

**Figure 1:**
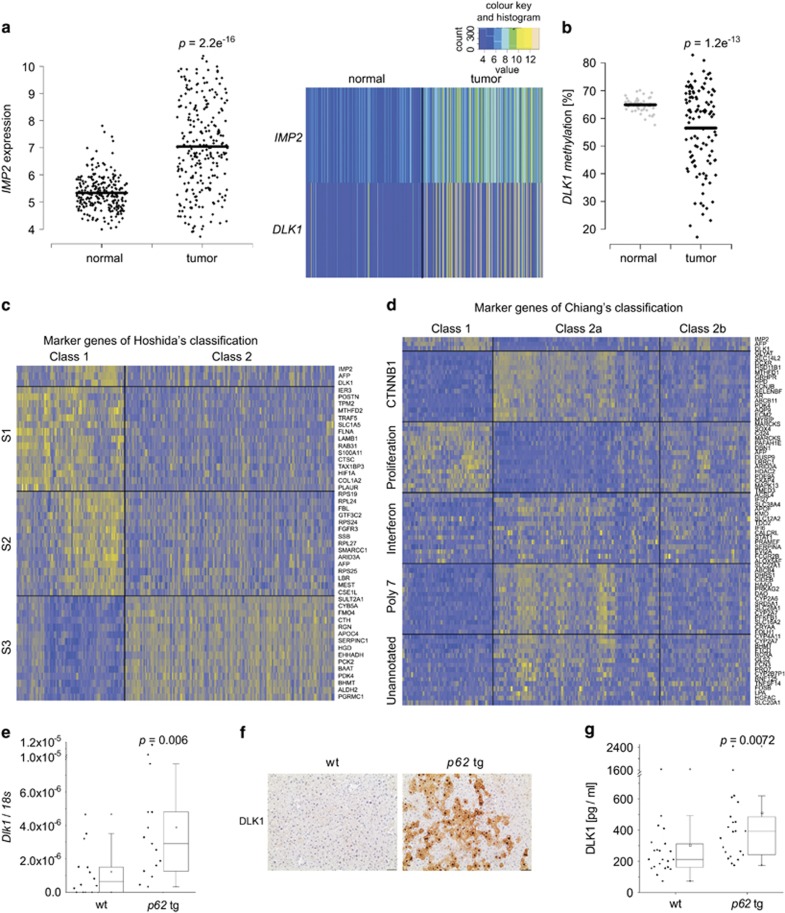
p62 and DLK1 expression in *p62* transgenic mice and human HCC. (**a**) Expression analysis of *IMP2* (left, right) and *DLK1* (right) in human HCC tumor (*n*=247) and normal liver (*n*=239) samples (GSE14520). (**b**) *DLK1* promoter methylation in human HCC tumor (*n*=109) and normal liver (*n*=50) samples (TCGA). (**c** and **d**) Heatmaps of clustering analyses according to Hoshida's (**c**) and Chiang's (**d**) HCC subsets.^17, 18^ The top 15 most differentially expressed genes are indicated for each class. (**e**) *DLK1* mRNA levels in livers of untreated animals 5 weeks of age: wt (*n*=14), *p62* transgenic (*p62* tg*; n*=15). Error bars show the interquartile range. (**f**) Representative immunohistochemical staining for DLK1 in untreated 5-week-old mice. Scale bars: 50 *μ*m. (**g**) Serum DLK1 protein levels in 5-week-old wt (*n*=22) and *p62* tg (*n*=22) mice. Error bars show the interquartile range

We investigated *IMP2* expression in a large patient cohort (GSE14520) of almost 250 predominantly hepatitis B virus (HBV)-positive HCC cases. *IMP2* was distinctly overexpressed in tumor compared with normal tissue (Figure 1a). *IMP2* strongly correlated with *α*-fetoprotein (*AFP*) as a marker of poor prognosis (*R*^2^=0.63; *P*<2.2e^−16^), which was also differentially expressed compared with normal tissue (*P*<2.2e^−16^). p62 was previously shown to induce the expression of the imprinted gene *IGF2*.^10, 13^ Another gene of the same imprinted gene cluster,^12^ *DLK1*, represents a marker of hepatic stem cells.^15^ *DLK1* was significantly overexpressed and its promoter was hypomethylated in human tumor tissue compared with normal samples (*P*=1.3e^−7^; Figure 1a and b) and strongly correlated with *IMP2* (*R*^2^=0.548; *P*<2.2e^−16^) and *AFP* (*R*^2^=0.535; *P*<2.2e^−16^).

In order to connect *IMP2*, *AFP*, and *DLK1* overexpression to already known molecular subsets of HCC,^16^ we performed hierarchical clustering of data set GSE14520 according to the marker genes identified by Hoshida *et al.*^17^ for three HCC subtypes in mouse and by Chiang *et al.*^18^ for five HCC subtypes in human (Supplementary Figure S1). Subsequent signal-to-noise ratio (SNR) analysis revealed that *IMP2*, *DLK1*, and *AFP* show similar expression patterns as marker genes of class 1, in which 84% of S1 and 65% of S2 marker genes were found (Supplementary Table S3). Class 2 can be described by subclass S3 presented by Hoshida *et al.* (Figure 1c, Supplementary Figure S3). Clustering by Chiang's marker genes resulted in three major classes (Supplementary Figure S2). Here, class 1, which included *IMP2*, *DLK1*, and *AFP*, was well related to Chiang's proliferation class. Class 2a can be described by elevated CTNNB1, Interferon, and Poly7 subclasses. Class 2b, however, was not related to any of Chiang's subclasses (Figure 1d, Supplementary Table S4,Supplementary Figure S3).

**Figure 2:**
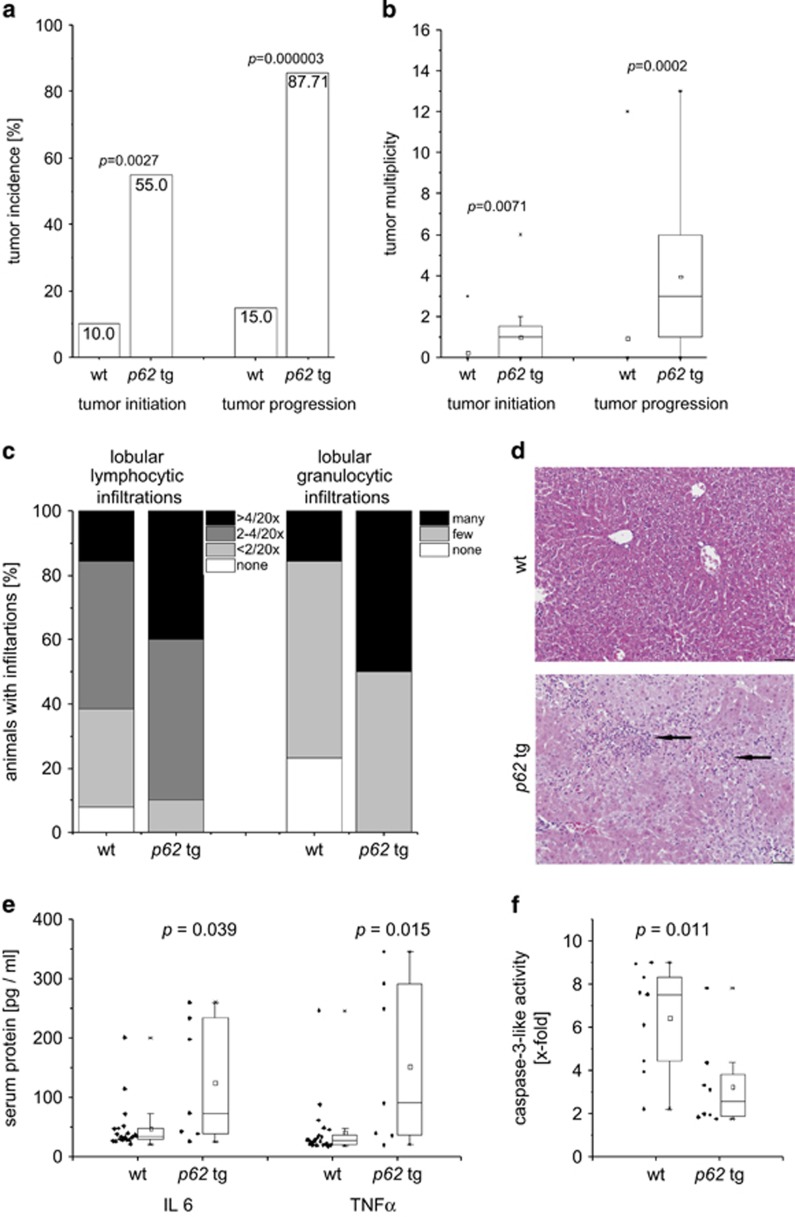
Susceptibility of *p62* transgenic mice to hepatocarcinogenesis. (**a** and **b**) Tumor incidence (**a**) and tumor multiplicity (**b**) in early stage (6 months: wt: *n*=20; *p62* tg: *n*=20; tumor initiation) and late stage (8 months: wt: *n*=20; *p62* tg: *n*=20; tumor progression) of DEN-treated mice. Error bars show the interquartile range. (**c** and **d**) Histological scoring of HE stainings and representative picture for lobular lymphocytic and granulocytic infiltrations 48  h after DEN application. Arrows denote mixed lymphocytic and granulocytic infiltrations. Scale bar: 50 *μ*m. (**e**) Serum protein levels of IL6 (left) and TNF*α* (right) of 5-week-old wt (*n*=22) and *p62* tg (*n*=7) mice 48 h after DEN application. (**f**) Caspase-3-like activity in DEN-treated 5-week-old wt (*n*=9) and p62 tg (*n*=8) mice 48 h after DEN injection normalized to untreated wt. Error bars show the interquartile range

A causal link of DLK1 expression to IMP2 was given by the fact that DLK1 mRNA and protein were increased in livers overexpressing the IMP2 splice variant p62 compared with wild types (wt; Figure 1e and f). Interestingly, also secreted DLK1 was elevated in the serum of *p62* transgenic animals (Figure 1g). As p62 induced the stem cell marker DLK1, we aimed to investigate the role of p62 in hepatocarcinogenesis employing the diethylnitrosamine (DEN) model. Both tumor incidence and tumor multiplicity were increased in DEN-treated *p62* transgenic animals during the early and late stages of tumor development (Figure 2a and b).

After 48 h of DEN treatment, which models early liver cell damage,^19^ *p62* transgenic mice revealed a more pronounced inflammatory response as shown by increased lobular lymphocytic as well as granulocytic infiltrations (Figure 2c and d) and by elevated serum levels of the inflammatory cytokines interleukin 6 (IL6) and tumor necrosis factor *α* (TNF*α*; Figure 2e). Neither AST nor ALT levels were different in *p62* transgenic animals compared with DEN-treated wt mice (Supplementary Table S5). Still, apoptosis was reduced in DEN-treated p62 transgenic animals (Figure 2f).

### Tumors of *p62* transgenic mice show a more aggressive phenotype

**Figure 3:**
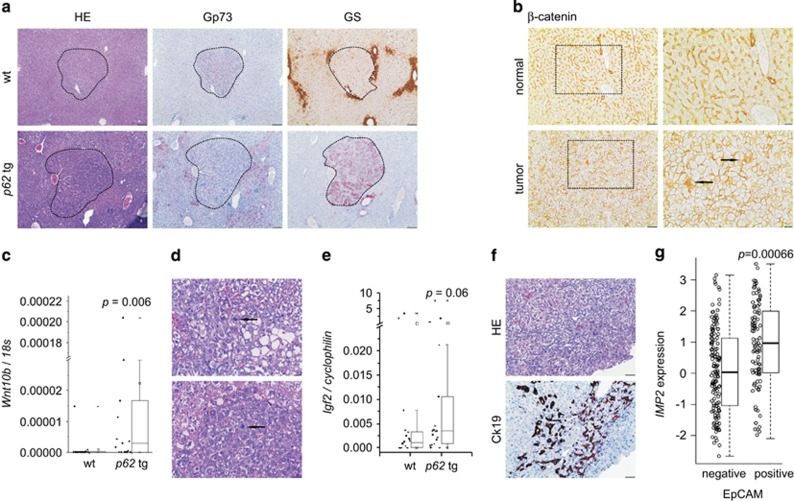
*p62* transgenic mice develop a more aggressive tumor phenotype. (**a**) Representative HE and immunostainings against Golgi membrane protein 73 (Gp73) and GS in wt and *p62* tg mice in late-stage tumors. Scale bars: 100 *μ*m. (**b**) Representative *β*-catenin immunostaining in adjacent normal and tumor tissue of livers bearing GS-positive tumors. Scale bars: 50 *μ*m (left), inset (right): 20 *μ*m. (**c**) *WNT10B* mRNA levels in wt (*n*=18) and *p62* tg (*n*=18) in the late tumor stage. Error bars show the interquartile range. (**d**) Arrows show irregular mitosis in representative HE stainings in tumors of *p62* tg mice. Scale bars: 20 *μ*m. (**e**) *Igf2* mRNA levels in wt (*n*=18) and *p62* tg (*n*=18) in the late tumor stage. Error bars show the interquartile range. (**f**) Representative HE and corresponding oval cell marker CK19 immunostaining in *p62* tg tumors. Scale bars: 50 *μ*m. (**g**) *IMP2* expression in human HCCs grouped into EpCAM-positive and -negative tumors (238 samples; GSE5975)

In order to characterize the DEN-induced tumors, paraffin sections were stained for the tumor markers Golgi membrane protein 73 (Gp73) and glutamine synthetase (GS). All wt tumors were Gp73-positive, whereas in transgenics only 70.31% were Gp73-positive. Interestingly, whereas none of the wt tumors stained positive for GS, 29.69% of p62 tumors were GS-positive and half of them were positive for both markers (Figure 3a). GS positivity is regarded as a marker of *β*-catenin activation,^20^ which can be regulated by activation of the canonical wingless-int (WNT) pathway. Concordantly, *β*-catenin staining confirmed its activation by nuclear and cytoplasmatic localization in tumor tissue, whereas normal tissue showed a membranous pattern (Figure 3b). *Wnt10b*, a canonical WNT pathway member, which is highly expressed in fetal, but shut down in adult liver, was increased in *p62* transgenic animals (Figure 3c). Tumors of transgenic animals were more mitotically active (*P*=0.0477) by irregular mitosis (Figure 3d) and were rather pleomorphic (0% in wt *versus* 15.6% in tg, *P*=0.014). mRNA levels of the pro-proliferative growth factor *Igf2* tended to be increased in *p62* transgenic animals (Figure 3e). CK19-positive oval cell compartments were solely observed in tumors of transgenics (Figure 3f). Concordantly, human HCCs positive for the oval cell marker EpCAM exhibited higher expression levels of *IMP2* compared with EpCAM-negative HCCs in an HBV-positive HCC cohort (238 samples; GSE5975; Figure 3g).

**Figure 4:**
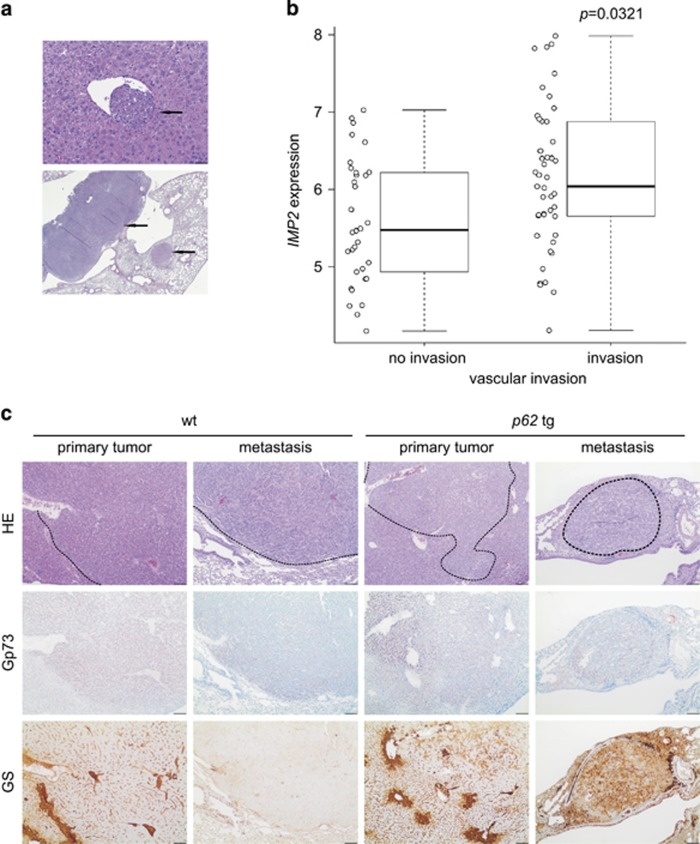
Metastasis in DEN-induced tumors. (**a**) Representative HE sections showing vascular invasion (upper panel, scale bar: 50 *μ*m) and lung metastases (lower panel, scale bar: 500 *μ*m). Arrows designate metastatic foci. (**b**) *IMP2* expression in human HCCs grouped into tumors positive or negative regarding vascular invasion (91 samples; GSE20238). (**c**) Representative HE and Gp73 and GS immunohistochemistry (IHC) of primary liver tumor and lung metastasis in wt (*n*=21) and *p62* tg (*n*=18) mice in the metastatic phase. Scale bars: liver: 200 *μ*m; lung: 100 *μ*m

Vascular invasion as well as lung metastases developed in both wt as well as in transgenic animals without statistically significant difference (Figure 4a). Analysis of a GEO data set of 226 predominantly viral hepatitis-related HCC cases (GSE20238) categorized by the presence or absence of vascular invasion revealed increased *IMP2* expression in patients with vascular invasion (Figure 4b).

**Figure 5:**
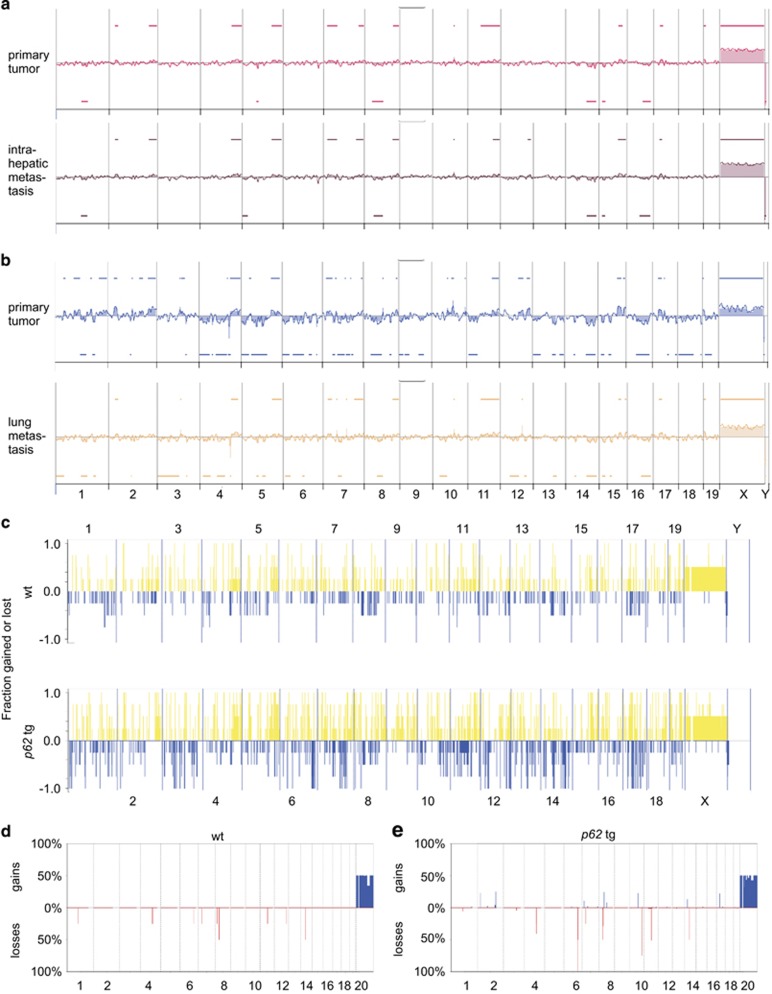
Clonality of primary tumors and metastases and genomic instability in tumors of *p62* transgenic mice. (**a** and **b**) Representative aCGH plots of primary HCC and corresponding intrahepatic metastasis (**a**) and of primary HCC and corresponding lung metastasis (**b**) of *p62* transgenic mice. (**c**) Frequency plot of fractions gained or lost along the genome of primary tumors in wt (*n*=4; upper panel) and *p62* tg (*n*=4; lower panel) mice in the late tumor stage. (**d** and **e**) Most significant alterations in primary tumors of wt (**d**) and *p62* tg (**e**) animals during the late tumor stage. Shown are percentages of gains and losses for individual altered segments obtained with the CGHcall package

Murine lung metastases showed the same staining pattern for the HCC markers GS and Gp73 as the primary liver tumors of wt and *p62* transgenic mice (Figure 4c). In the metastatic phase, also some wt tumors showed positive GS staining (data not shown). aCGH analysis confirmed clonality of primary tumors and both intrahepatic (*P*<10^−5^) as well as lung metastases (*P*<10^−5^; Figure 5a and b, Supplementary Table S6).

### *p62* transgenic mice are more susceptible to chromosomal aberrations

aCGH analysis (Figure 5c) revealed increased alterations in tumors of transgenic (lower panel) compared with wt animals (upper panel). Significant gains were only observed in transgenic tumors, and significant losses were stronger in transgenic compared with wt tumors (Figure 5d and e). Some loci only showed aberrations in *p62* transgenic mice: their corresponding loci on the human genome are given in Supplementary Table S7. Gene Ontology analysis revealed that the affected loci harbor genes, which are involved in growth, proliferation, negative apoptosis signaling, and angiogenesis (Supplementary Table S8). Interestingly, the distal mouse 15B3.1-C region, amplified only in *p62* transgenics and corresponding to the human distal chromosome 8q23.1-23.3, is the second most frequently amplified region in human HCC: aCGH results from 848 HCC samples show an amplification in ~45% of cases (www.progenetix.net). This region comprises genes commonly mutated in cancer (Supplementary Table S7).

### Tumor-promoting DLK1 drives RAC1-induced ROS formation

**Figure 6:**
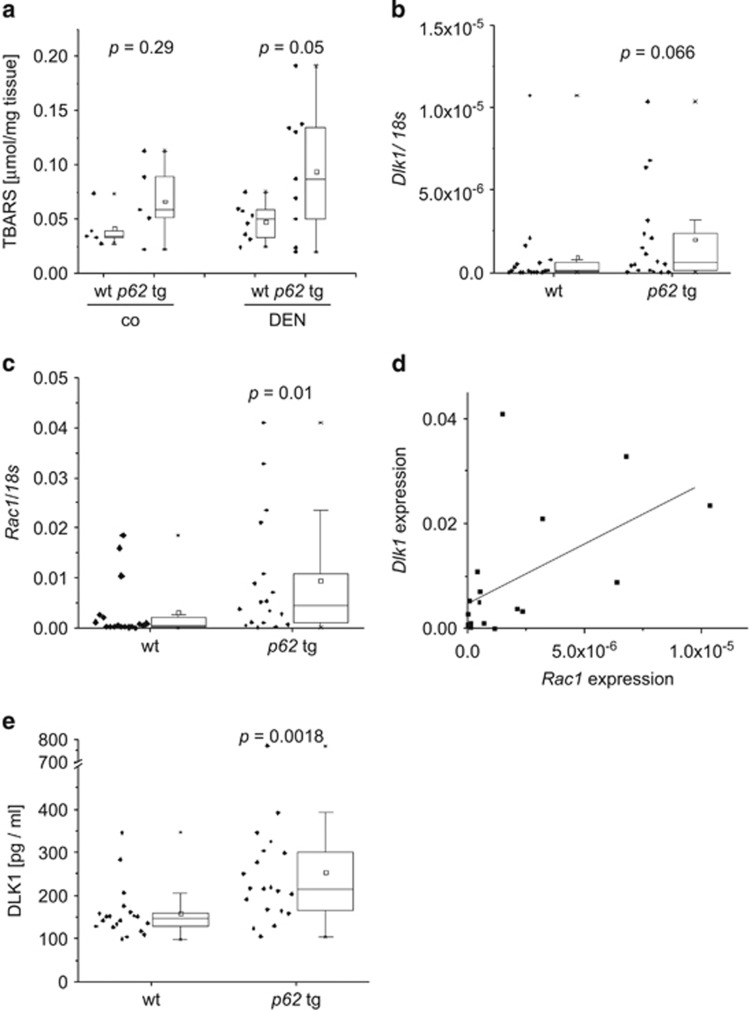
Increased levels of ROS and ROS-inducer RAC1 in *p62* transgenic mice. (**a**) Hepatic TBARS levels in wt (*n*=5) and *p62* tg (*n*=5) livers of untreated (co) and 48-h DEN-treated (DEN) animals (wt: *n*=8; tg: *n*=9). Error bars show the interquartile range. (**b**–**d**) *Dlk1* (**b**) and *Rac1* (**c**) mRNA expression as well as correlation of both **d** was investigated after 8 months (wt: *n*=18; tg:*n*=18). Error bars show the interquartile range. (**e**) Secreted DLK1 protein serum levels were measured by ELISA. Error bars show the interquartile range

**Figure 7:**
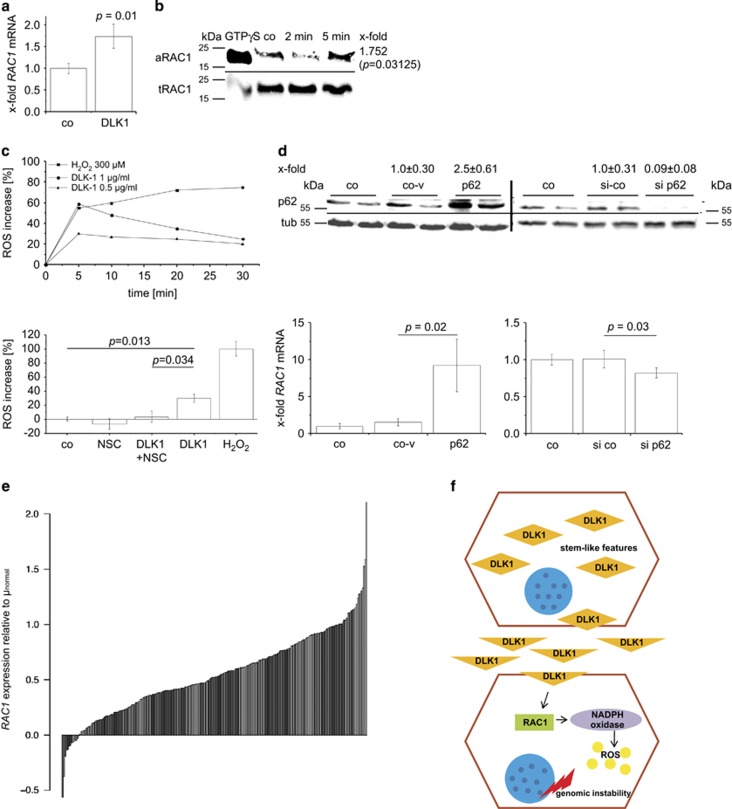
p62 promotes DLK1-RAC1-driven ROS generation. (**a** and **b**) Levels of *RAC1* mRNA presented as mean±S.E.M. (**a**) and activated RAC1 protein levels determined by pull-down assay (**b**) in HepG2 cells after treatment with 1 *μ*g/ml DLK1 protein (*n*=3 in duplicate). (**b**) Representative pull-down assay with activated RAC1 (aRAC1) and total RAC1 (tRAC1) is shown. X-fold signal intensities of 5-min treatment with DLK1 were normalized to untreated control (co). (**c**) ROS levels: representative experiment (quintuplicates) of HepG2 cells treated with 0.5 or 1 *μ*g/ml DLK1 or H_2_O_2_ as positive control for 0–30 min (upper part). Data are normalized to untreated HepG2 cells. ROS levels in HepG2 cells treated with either DLK1 or RAC1 inhibitor NSC23766 alone or in combination (lower part). Untreated HepG2 cells served as control. H_2_O_2_-induced ROS formation was set to 100% (*n*=2, quintuplicate). Data are presented as mean±S.E.M. (**d**) *RAC1* expression in HepG2 cells overexpressing (left) p62 sense plasmid (p62) compared with antisense-plasmid (co-v), untreated control (co), and siRNA knockdown (right) of p62 (si p62) compared with random siRNA (si co; *n*=3 triplicate/quadruplicate). Data show mean±S.E.M. Western blot knockdown/overexpression control was densitometrically quantified (*n*=4 triplicate/quadruplicate; upper part). (**e**) *RAC1* expression in human HCC (GSE14520) normalized to the mean of normal samples. (**f**) Overview of p62-promoted DLK1-RAC1-induced genomic instability. DLK1-overexpressing cells with stem-cell-like features secrete DLK1 protein, which activates RAC1 in a paracrine manner, in turn leading to ROS generation via NADPH oxidase. Elevated ROS levels finally result in genomic instability

We sought to identify the reason for p62-induced increased genomic instability and found significantly elevated levels of TBARS as indicators of oxidant stress in *p62* transgenic animals after short-term treatment with DEN (Figure 6a). ROS are important inducers of DNA damage and chromosomal instability.^2^ NADPH oxidase represents an ROS-generating enzyme complex that contributes to DEN-induced carcinogenesis.^21^ NADPH oxidase is activated by the small GTPase RAC1,^22^ and DLK1 was previously shown to induce RAC1.^23^ We observed increased levels of both *Dlk1* and *Rac1* mRNA in *p62* transgenic livers and a strong correlation between each other (Pearson *R*^2^=0.56, *P*=0.015; Figure 6b–d). The secreted form of DLK1 was elevated in *p62* transgenic mice (Figure 6e). In order to test the causal effect of p62 and DLK1 on RAC1, *in vitro* experiments were performed. DLK1 treatment increased *RAC1* mRNA levels as well as activated RAC1 protein as detected by pull-down assay in HepG2 cells (Figure 7a and b). Furthermore, DLK1 treatment increased ROS levels, which was completely abrogated by pre-incubation with the RAC1 inhibitor NSC23766 (Figure 7c). In addition, p62 overexpression increased *RAC1* expression (Figure 7d). *Vice versa*, knockdown of p62 led to decreased *RAC1* mRNA levels (Figure 7d). ROS levels were elevated after p62 overexpression by 9.46±1.24% (*P*=0.045) 48 h after transfection, which was abrogated by the RAC1 inhibitor (*P*=0.0046). Finally, the human HCC cohort, which showed differential expression of *IMP2* and *DLK1* (Figure 1a), significantly overexpressed *RAC1* (Figure 7e; *P*<2.2e^−16^). SNR analysis revealed *RAC1* overexpression in class 1 (Supplementary Tables S3 and S4), which was characterized by *IGF2BP2*, *AFP*, and *DLK1* overexpression (Figure 1b).

## Discussion

The IMP p62 was originally identified as a tumor-associated autoantigen with autoantibodies against p62 detected in HCC patients^4^ and in several other types of cancer.^24, 25^ Interestingly, despite several investigations of p62 autoantibodies as a potential tumor marker and a recently suggested resistance of *IMP2* knockout mice toward malignancy,^26^ functional implications of the p62 protein in carcinogenesis are widely unknown. Our analysis of a large homogenous human HCC cohort with ~250 viral HCC samples showed strongly increased expression of *IMP2* in the majority of HCC patients. These data are supported by other reports suggesting elevated levels of p62 in HCC tissue in rather small patient cohorts.^13, 27^ According to the classification performed in this study, overexpression of *AFP* and *IGF2*, both correlating with *IMP2* expression in HCC (present findings and Kessler *et al.*^13^), marks Hoshida's S2 class of aggressive HCC.^28^ Positivity of the stem cell surface antigen EpCAM and vascular invasion, which we observed to be linked to *IMP2* overexpression, was used as a classification system by others.^29, 30^ In fact, EpCAM expression is associated with early recurrence and short survival time.^31^

Regarding the classification from Boyault *et al.*,^32^ *IMP2*-overexpressing samples probably belong to the G1 subset, which is characterized by an increased expression of *AFP* and the imprinted gene products *IGF2* and *H19*. *p62* transgenic mice were shown to overexpress both imprinted genes.^10^ Finally, *IMP2*-overexpressing samples match the molecular pattern of Cairo's aggressive hepatoblastoma, in which *AFP*, *Krt19*, and *EpCAM* are elevated. In the same study the authors provide data from Myc-induced murine tumors highly expressing *DLK1*, *IGF2*, and *AFP*.^33^ A summary of assignments of class 1 and class 2 HCCs to known molecular HCC subsets is given in Supplementary Figure S4.

Interestingly, we observed a correlation of *IMP2* expression with the oval/stem cell marker *DLK1*.^15^ DLK1 was previously shown to correspond with poor survival in HCC.^34^ Oval cells share phenotypic markers with embryonic hepatoblasts, in which DLK1 is also highly expressed.^35^ The cytoplasmatic appearance of DLK1 in *p62* transgenic mice reveals a fetal phenotype as previously reported for HCC and hepatoblastoma tissue.^36^ Secreted DLK1, suggested as a serum marker for hepatoblastoma,^37^ was elevated in sera of *p62* transgenic mice.

Secreted DLK1 was suggested to have paracrine functions, that is, inducing the secretion of inflammatory cytokines, such as TNF*α* and IL6 in monocytes and adipocytes.^38^ Recently, p62 expression was shown to promote liver disease by amplifying inflammatory processes.^9, 11, 39, 40, 41^ HCC mostly develops within an inflammatory environment, such as viral hepatitis, ASH and NASH, and inflammatory mediators promote hepatocarcinogenesis.^19^ We here present a transgenic mouse model, which develops HCC out of an inflammatory state involving elevated IL6 and TNF*α* productions. We observed an early onset and an accelerated progression of HCC in *p62* transgenic mice.

There are two different models using the carcinogen DEN to induce liver tumors. DEN is either given as a single dose by itself or in combination with the tumor-promoting agent phenobarbital to induce tumors with *β*-catenin mutations, which are linked to GS positivity.^20^ Interestingly, employing *p62* transgenic mice, we observed GS-positive tumors in the DEN model without using phenobarbital.

The expression of DLK1 is closely linked to WNT10B, a member of the canonical WNT pathway, leading to *β*-catenin accumulation in the cytoplasm and the nucleus, which can be altered by DLK1.^42^ Both elevated *Wnt10b* and cytoplasmatic/nuclear localization can be found in *p62* transgenic tumors.

In tumors positive for the stem cell marker EpCAM, co-expression of DLK1 and AFP was defined by poor prognosis.^43^ Tumors of *p62* transgenic livers were more susceptible to chromosomal aberrations than tumors of wt animals and showed more pronounced alterations. Increasing levels of chromosomal instability correlate with progression of HCC, suggesting that marked genomic instability characterizes more advanced stages of the disease. The homolog of human 8q23, amplified specifically in *p62* transgenic animals, is frequently gained in human HCC tissues.^44^ Interestingly, amplification of the homolog of human chromosome 3q, which was gained in *p62* transgenic tumors, is correlated with advanced-stage disease in cervical carcinomas.^45^ The losses specifically observed in *p62* transgenics on the homologs of human chromosomes 9q33.3-34.3, 11q23.1-24.1, 16q42.13-42.2, and 21q22.11-3 were reported to be deleted in different types of cancer including HCC.^46, 47^

Genomic instability can be induced through ROS production.^2^ A major ROS-generating enzyme complex, the NADPH oxidase, is activated by the small GTPase RAC1.^22^ We found *RAC1* to be highly overexpressed in a large proportion of HCC tissues. RAC1 itself has been described to have a role in HCC^48^ and might act at least partly via ROS production.^49^ In addition, Ras-induced ROS production and DNA damage has been linked to RAC1 activation.^50^ Our data functionally link the aggressive and dedifferentiated phenotype of the tumors in *p62* transgenic livers to DLK1-facilitated induction of RAC1. The stem cell marker and paracrine factor DLK1 was previously reported to induce RAC1 activation in 3T3-L1 cells.^23^ We here report that DLK1-induced RAC1 activation leads to elevated ROS levels (Figure 7f). We suggest that the DLK1/RAC1-induced increase in ROS is the cause of chromosomal instability,^2^ which in turn leads to more undifferentiated tumors.^51^ Interestingly, RAC1 activation was shown to drive proliferation of intestinal stem cells^52^ and targeting RAC1 suppresses cancer cell viability,^53^ cancer stem cell activities,^54^ and metastasis.^55^ Wang *et al.*^56^ reported that RAC GTPase-activating protein 1 is associated with early recurrence in HCC.

Taken together, our *in vivo*, *in vitro*, and *in silico* analyses show that IMP2/p62 has an important role in HCC initiation and progression and characterizes human HCC prognosis.

## Materials and Methods

### Animals

All animal procedures were performed in accordance with the local animal welfare committee. Mice were kept under controlled conditions regarding temperature, humidity, 12 h day/night rhythm, and food access. *p62* transgenic mice expressing the transgene exclusively in the liver were established as previously described.^10^ For the short-term experiment, *p62* transgenic (*p*62 tg) mice and matched wt littermates were treated with 100 mg/kg body weight (BW) DEN i.p. at the age of 2.5 or 5 weeks and were killed 48 h later.^19, 39^ For tumor induction, *p62* transgenic mice and wt littermates were injected with 5 mg/kg BW at the age of 2 weeks. In long-term experiments, mice were killed at an age of 6 and 8 months modeling an early (tumor initiation) and late (tumor progression) tumor stage, respectively.^57^ Metastases were investigated in animals older than 10 months (metastatic phase; wt: *n*=21, tg: *n*=18).

### Real-time quantitative polymerase chain reaction

Experiments and quantification were performed as described in detail previously.^9^ Sequences and conditions are given in Supplementary Table S1.

### ELISA

Serum levels of IL6 (#m6000b, R&D Systems, Wiesbaden, Germany), TNF*α* (#mta00b, R&D Systems), and DLK1 (#CSB-EL006945MO, Cusabio Biotech, Wuhan, China) were performed with ELISA according to the manufacturers' instructions.

### Immunohistochemistry

Demasking, antibody dilutions, and detection reagents are given in Supplementary Table S2. Primary antibodies used were specific to GS, Golgi membrane protein 73 (Gp73), *β*-catenin, and p62.^13^ Samples were examined by two independent investigators blinded to experimental conditions.

### Western blot

Western blot analysis of p62 protein levels was performed according to Kessler *et al.*^13^

### Quantification of thiobarbituric acid-reactive substances

Products of lipid peroxidation were measured as previously described.^40^

### Cell culture

Knockdown and overexpression experiments for p62 in HepG2 were performed as previously described.^13^ p62 sense and antisense constructs are available at Addgene (#42174 and #42175, Cambridge, MA, USA). Recombinant DLK1 was used for treatment (#1144-PR-025, R&D Systems).

### Caspase-3-like activity assay

Caspase-3-like activity assay was perfomed as previously described.^13^ The extraction buffer was slightly modified: 25 mM HEPES pH 7.5, 5 mM MgCl_2_, 1 mM EGTA, pepstatin, leupeptin, and aprotinin (1 *μ*g/ml each).

### ROS assay

ROS assay was performed as previously described.^22^ HepG2 cells were loaded with either 20 *μ*M 2′,7′-dichlorodihydrofluorescein diacetate alone or with the RAC1 inhibitor NSC23766 (#2161/10, R&D Systems) in PBS 60 min before DLK1 treatment for 48 h after transfection and 50 min before measurement, respectively. Combined DLK1 and NSC23766 treatment was performed for 5 min. DLK1/H_2_O_2_ (positive control) treatment over time (5–30 min) was performed in quintuplicates.

### RAC1 pull-down assay

Activated RAC1 levels were measured by pull-down assay as previously described.^22, 58^

The affinity precipitation assay detects the binding of active RAC1 to a fusion protein consisting of the RAC1 target p21-activated kinase 1 and glutathione S-transferase (GST). GST-PBD was expressed in *Escherichia coli*, purified, and bound to glutathione Sepharose beads (#17-0756-01, GE Healthcare Life Sciences, Freiburg, Germany). For RAC1 pull-down assays, HepG2 cells were treated with 1 *μ*g/ml recombinant DLK1 (#1144-PR-025, R&D Systems), cells were washed with ice-cold PBS, and were lysed with PBD-buffer (Tris pH 8.0 25 mM, DTT 1 mM, MgCl_2_ 20 mM, NaCl 100 mM, EDTA 0.5 mM, Triton X-100 1%, Aproptinin 0.1%, Leupeptin 0.1%, and PMSF 0.1%). As a positive control, one sample was lysed with GTP*γ*S-PBD-buffer (Tris pH 8.0 25 mM, DTT 1 mM, MgCl_2_ 5 mM, NaCl 100 mM, EDTA 1 mM, Triton X-100 1%, Aprotinin 0.1%, Leupeptin 0.1%, and PMSF 0.1%). After scraping cells off, cells were incubated for complete lysis for 15 min at 4 °C under vigorous shaking. The positive control was incubated for 10 min with GTPγS (10 mM), leading to an exchange of RAC-GDP to RAC-GTP, which was stopped by adding MgCl_2_ (1 M). After centrifugation, the supernatants of cell lysates and positive control were incubated with 30 *μ*l GST-PBD beads for 2 h at 4 °C under vigorous shaking. After centrifugation and one wash step with PBD-/GTP*γ*S-PBD-buffer, the pellet was frozen at −80 °C.

Pull-down supernatants and pellets with loading buffer were boiled for 10 min. Subsequently, the samples were separated using SDS-PAGE on 12% gels and are transferred onto Immobilon-FLPVDF membranes (Rockland, Gilbertsville, PA, USA). The membranes were blocked and incubated with primary antibody overnight at 4 °C, followed by incubation with IRDye-conjugated secondary antibody. After washing, blots were scanned with an Odyssey Infrared Imaging System (LI-COR Biotechnology, Bad Homburg, Germany) and signal intensities were determined using the Odyssey software.

### aCGH analysis

Paraffin-embedded liver tumors were microdissected and hybridized against 3-month-old wt liver tissues. Labeling was performed following the BioPrime aCGH Genomic Labeling Module protocol (Invitrogen, ThermoFisher, Dreieich, Germany). The samples were hybridized on an 8x60-k CGH Array under the conditions of the Agilent protocol (Version 7.2). The arrays were analyzed with an Agilent DNA Microarray Scanner G2505C (Agilent, Böblingen, Germany) and the extraction software Agilent Feature Extraction 11.0.1.1. The data were analyzed using the statistical software R Bioconductor packages aCGH^59^ and CGHcall.^60^ In order to compute the similarity of aberrations in the primary tumor and the corresponding metastasis, permutation tests were used to calculate the pair-wise statistical significance similar to the method described in Haybaeck *et al.*^61^ Aberrations were labeled using the bioconductor package aCGH with standard log ratio threshold of |0.25|.^59^ The number of matching positions was calculated in the two samples. The aberration positions of the sample containing fewer aberrations were randomly reordered, matched to a random set of aberration positions of the other sample, and the new number of matching positions (ri) was calculated. This step was repeated *n*=100 000 times and the number of times *r* that showed a higher number of matching aberrations of the randomly reshuffled samples compared with the original samples was counted as *r*=sum (ri> o). The *P-*value for the statistical significance of matching positions of gains or losses was estimated as *P*=*r*/*n*. Overlap of aberrations and *P-*values of similarity are provided in Supplementary Table S6. Locations of aberrations specifically observed in the *p62* transgenic animals were detected using the Golden Helix software: analysis was conducted using SNP an Variation Suite v8. These loci were compared with the aberrant loci of human HCC samples on www.progenetix.org. GOSim was used to identify enriched Gene Ontology terms.^62^ The mutation data were obtained from the Sanger Institute COSMIC website, http://www.sanger.ac.uk/cosmic. In addition, the CGHcall package^60^ was used to search for significant alterations. CGHcall employs DNAcopy methods^63^ to normalize and smoothen the data and defines equal copy number segments for further analysis.

### Human GEO data sets

For differential gene expression between tumor (*n*=247) and non-tumor (*n*=239) samples, the log2 of an RMA-normalized data set (GSE14520)^64^ of an AffymetrixGeneChip HG-U133A 2.0 was analyzed. Similarly, differential gene expression was analyzed in data set GSE5975 between positive (*n*=95) and negative (*n*=143) EpCAM samples and in data set GSE20238 between vascular invasive (*n*=45) and non-invasive (*n*=34) HCC samples. Differential expression analysis was based on the Kolmogorov–Smirnov test. Pearson correlation was applied to detect correlations between genes of interest.

### Identification of common molecular HCC subclasses

Complete hierarchical clustering of data set GSE14520^64^ was performed using the marker genes presented by Hoshida *et al.*^17^ and Chiang *et al.*^18^ The cluster dendograms are provided below Supplementary Figure S1. To test the affiliation of genes with HCC subtypes, the SNR was calculated for each marker gene as described in Hoshida *et al.*^17^ and Golub *et al.*^65^

### Methylation analysis using a TCGA data set

TCGA analysis of DNA methylation in HCC was performed using an Illumina Infinium Human Methylation 450 platform. The data set contains 50 normal and 109 tumor samples. We considered methylation only in the promoter regions (defined within 2000 bp from the transcription start site provided in the EPD promoter DB.^66^ Averages were considered for regions covered by multiple probes.

### Statistical analysis

Data analysis and statistics of experimental data were performed using the Origin software (OriginPro 8.1G; OriginLabs, Northampton, MA, USA). All data are displayed either as columns with mean values±S.D. or as individual values and boxplots±interquartile range with mean and median. Statistical differences were estimated by independent two-sample *t*-test or Wilcoxon-rank-sum test depending on normal distribution, which was tested by the Shapiro–Wilk method, or Fisher-exact test for categorical data. Normally distributed data comparing multiple groups were analyzed using ANOVA combined with Bonferroni *post hoc* test. All tests are two-sided, and differences were considered statistically significant when *P-*values were less than 0.05.

## Abbreviations

HCC: hepatocellular carcinoma
IGF2: insulin-like growth factor 2
IMP2-2/IGF2BP2-2: insulin-like growth factor 2 mRNA-binding protein
BW: body weight
DEN: diethylnitrosamine
TBARS: thiobarbituric acid-reactive substances
GS: glutamine synthetase
Gp73: Golgi membrane protein 73
HBV: hepatitis B virus
ROS: reactive oxygen species
TNF*α*: tumor necrosis factor*α*
IL6: interleukin 6
DLK1: delta-like 1 homolog
GST: glutathione S-transferase
SNR: signal-to-noise ratio
WNT: wingless-int
*AFP*: *α*-fetoprotein
EpCAM: epithelial cell adhesion molecule
