# Supplementary material for: IMP2/p62 induces genomic instability and an aggressive hepatocellular carcinoma phenotype
Source: Cell Death Dis. 2015 Oct 1;6(10):e1894–. doi: 10.1038/cddis.2015.241 (PMC4632283; doi:10.1038/cddis.2015.241)
Supplement: Supplementary Information [file cddis2015241x1.docx]

**Supplementary Material**

**IMP2/p62 INDUCES GENOMIC INSTABILITY AND AN AGGRESSIVE HCC PHENOTYPE**

Sonja M. Kessler^1,2^, Stephan Laggai^1^, Ahmad Barghash^3,5^, Christina S. Schultheiss^1^, Eva Lederer^2^, Monika Artl^4^, Volkhard Helms^3^, Johannes Haybaeck^2^, Alexandra K. Kiemer*^1^

^1^Department of Pharmacy, Pharmaceutical Biology, Saarland University, Saarbruecken, Germany

^2^Institute of Pathology, Medical University of Graz, Austria

^3^Center for Bioinformatics, Saarland University, Saarbruecken, Germany

^4^Institute of Human Genetics, Medical University of Graz, Austria

^5^Saarbruecken Graduate School of Computer Science, Saarbruecken, Germany

*corresponding author

**Table of contents**

Supplementary Figures 3

Figure S1 3

Figure S2 4

Figure S3 5

References Figure S3 6

Supplementary Tables 7

Table S1 7

Table S2 8

Table S5 9

Table S6 10

Table S8 11

**Supplementary Figures: Figure S1**

**
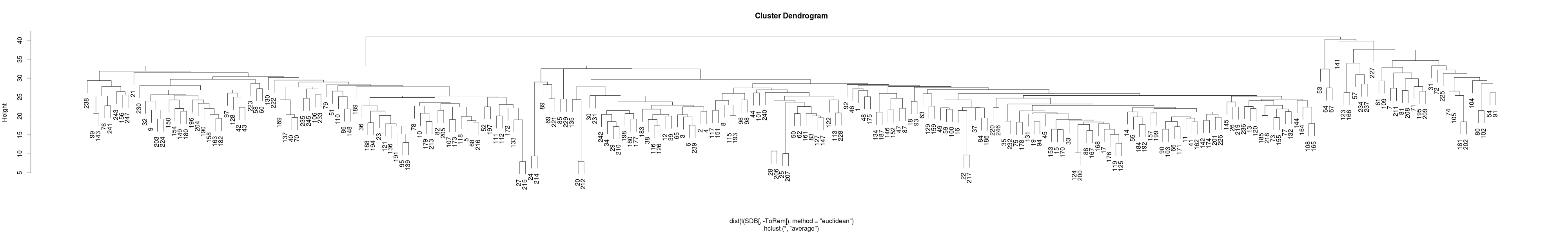
**

Fig. S2: Cluster dendogram of complete hierarchical clustering analysis of dataset GSE14520 using marker genes presented by Hoshida et al. (1). Two major subclasses were identified.

**Figure S2**

**
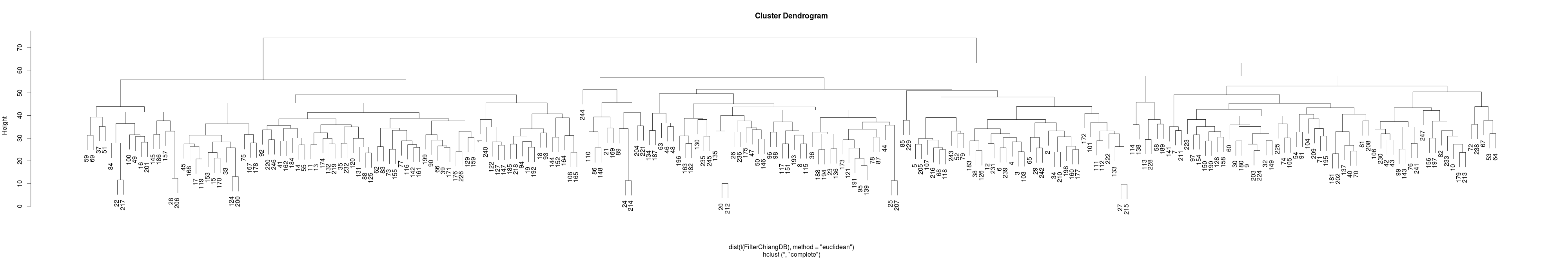
**

Fig. S3: Cluster dendogram of complete hierarchical clustering analysis of dataset GSE14520 using marker genes presented by Chiang et al. (2). Three major subclasses were identified.

**Figure S3**

**IMP2↑, AFP↑, DLK1↑**

aggressive HCC

**Class 1**: **IMP2↑**

less-aggressive HCC

**Class 2**

**Class 2a Class 2b**

S1 / S2 [1]

S3 [1]

Proliferation [2]

EpCAM+ [3]: IMP2↑

Vascular invasion [4]: IMP2↑

Aggressive hepatoblastoma: AFP↑,

Myc-induced liver tumors: AFP↑, DLK1↑, IGF2↑ [6]

CTNNB1, Interferon,

Polysomy 7 [2]

G1: AFP↑, IGF2↑ [5]

Clustering Hoshida et al.

Clustering Chiang et al.

Analysis of expression

Literature- based signatures

Fig. S3: Overview of molecular subclasses of hepatocellular carcinoma (HCC) focusing on HCC overexpressing *IMP2*, *AFP*, and *DLK1*. Correspondence between class 1 and known subclasses definded by clustering and subsequent SNR analysis (upper panel). Correlation of known classifications and IMP2 expression evaluated by gene expression analysis (middle panel). Signatures/subclasses characterized by marker genes in the literature (lower panel).

References

[1] Hoshida Y, Nijman SMB, Kobayashi M, Chan JA, Brunet JP, Chiang DY, Villanueva A, et al. Integrative transcriptome analysis reveals common molecular subclasses of human hepatocellular carcinoma. Cancer Res 2009;69:7385.

[2] Chiang DY, Villanueva A, Hoshida Y, Peix J, Newell P, Minguez B, LeBlanc AC, et al. Focal gains of VEGFA and molecular classification of hepatocellular carcinoma. Cancer Res 2008;68:6779.

[3] Yamashita T, Forgues M, Wang W, Jin WK, Ye Q, Jia H, Budhu A, et al. EpCAM and alpha-fetoprotein expression defines novel prognostic subtypes of hepatocellular carcinoma. Cancer Res 2008;68:1451.

[4] Mínguez B, Hoshida Y, Villanueva A, Toffanin S, Cabellos L, Thung S, Mandeli J, et al. Gene-expression signature of vascular invasion in hepatocellular carcinoma. J Hepatol 2011;55:1325.

[5] Boyault S, Rickman DS, De Reyniès A, Balabaud C, Rebouissou S, Jeannot E, Hérault A, et al. Transcriptome classification of HCC is related to gene alterations and to new therapeutic targets. Hepatology 2007;45:42.

[6] Cairo S, Armengol C, De Reynias Al, Wei Y, Thomas E, Renard C-Al, Goga A, et al. Hepatic Stem-like Phenotype and Interplay of Wnt/b-Catenin and Myc Signaling in Aggressive Childhood Liver Cancer. Cancer Cell 2008;14:471.

**Supplementary Tables**

**Table S1** *Primer and conditions*

| **gene** | **forward primer sequence**  **5´-3´** | **reverse primer sequence**  **5´-3´** | **amplicon size**  **[bp]** | **gene ID** | **primer**  **[nM]** | **annealing temp. [°C]** |
| --- | --- | --- | --- | --- | --- | --- |
| hu ACTB | TGCGTGACATTAAGGAGAAG | GTCAGGCAGCTCGTAGCTCT | 107 | NM_001101 | 200 | 60 |
| hu p62 | GTTCCCGCATCATCACTCTTAT | GAATCTCGCCAGCTGTTTGA | 117 | AF057352 | 200 | 62 |
| hu RAC1 | AAGAGAAAATGCCTGCTGTTGTAA | GCGTACAAAGGTTCCAAGGG | 72 | NM_006908.4 | 200 | 60 |
| mu 18s | GTAACCCGTTGAACCCCATT | CCATCCAATCGGTAGTAGCG | 151 | NR_003278.1 | 200 | 58 |
| mu Dlk1 | ACTTGCGTGGACCTGGAGAA | CTGTTGGTTGCGGCTACGAT | 221 | NM_010052.5 | 200 | 58 |
| mu Rac1 | GCGAAAGAGATCGGTGCTGT | GACAGAGAACCGCTCGGATAG | 100 | NM_009007.2 | 200 | 62 |
| mu Wnt10b | ATCGCCGTTCACGAGTGTC | GGAAACCGCGCTTGAGGAT | 111 | NM_011718.2 | 200 | 61 |

**Table S2** *Antibodies and conditions*

| **antibody** | **product no.** | **company** | **demasking** | **concentration** | **incubation** | **detection** |
| --- | --- | --- | --- | --- | --- | --- |
| anti-DLK1 | ab21682 | Abcam | Citrate buffer pH 6.0, 30 min, waterbath 100 °C | 1:1,000 | 4 °C, overnight | Dako Envision DAB |
| anti-Gp73 | sc-48011 | Santa Cruz | Citrate buffer pH 6.0, microwave | 1:200 | RT, 60 min | rabbit-anti-goat (#A50-204A, Bethyl), Dako Envision DAB |
| anti-Glutamin synthetase | MAB3002 | Millipore | CC1 mild (Ventana), 30 min | 1:5,000 | RT, 30 min | Ultra View (Ventana) DAB or AEC |
| anti-beta-Catenin | D10A8 | Cell Signaling Technology | Citrate buffer pH 6.0, 30 min, waterbath 100 °C | 1:1,000 | 4 °C overnight | Dako Envision DAB |
| anti-CK19 | 3863-S | Epitomics | Epitope retrieval (Dako #K5207), 40 min, waterbath 100 °C | 1:500 | RT, 60 min | Dako Envision AEC |

**Table S5** *Serum parameters*

Table shows mean serum levels ± SEM of 5 week-old wt (n=25) or tg (n=12) animals 48 h after DEN treatment.

|  | **wt** | **tg** | ***p*** |
| --- | --- | --- | --- |
| **AST [U/l]** | 3,283 ± 253 | 3,619 ± 342 | 0.45 |
| **ALT [U/l]** | 517 ± 50 | 591 ± 66 | 0.33 |
| **Glucose [mg/dl]** | 151 ± 5.5 | 97.6 ± 10.6 | 0.00027 |
| **Triglcerides [mg/dl]** | 163 ± 13.3 | 114 ± 10.0 | 0.025 |
| **HDL [mg/dl]** | 88.5 ± 4.7 | 128 ± 13.5 | 0.012 |
| **Cholesterol [mg/dl]** | 116 ± 4.8 | 160 ± 13.7 | 0.0097 |

**Table S6** *Clonality of primary tumors and metastases*

Table shows overlap of aberrations and corresponding *p*-value of primary hepatic tumor and metastasis.

| Sample 1 | Sample 2 | Aberrations sample 1 | Aberrations sample 2 | Common no aberrations | Common gains | Common losses | Above threshold | *p*-value |
| --- | --- | --- | --- | --- | --- | --- | --- | --- |
| 1136_3_M1 | 1136_3_T1 | 4471 | 7724 | 45446 | 1533 | 1279 | 0 | <1e-5 |
| 1138_9_1_M1 | 1138_9_1_T1 | 1948 | 2828 | 51423 | 900 | 470 | 0 | <1e-5 |
| 1138_9_5_M1 | 1138_9_5_T1 | 2149 | 4452 | 49916 | 1055 | 630 | 0 | <1e-5 |
| 985_1_M1 | 985_1_T1 | 5865 | 2553 | 48440 | 1359 | 670 | 0 | <1e-5 |
| 987_1_T1 | 987_1_T2 | 2905 | 2617 | 51417 | 1393 | 717 | 0 | <1e-5 |
| 987_2_T1 | 987_2_T2 | 2506 | 2376 | 51924 | 1279 | 698 | 0 | <1e-5 |
| 991_2_T5 | 991_2_T7 | 831 | 1828 | 52836 | 446 | 220 | 0 | <1e-5 |

**Table S8** *GO terms of human loci corresponding to aberrant loci in p62 transgenic mice*

| **corresponding**  **human locus** | **go_id** | **term** | **p-value** |
| --- | --- | --- | --- |
| Chr5p15.1‐14.1 | GO:0007155 | cell adhesion | 0.006449712 |
|  | GO:0006637 | acyl‐CoA metabolic process | 0.002774436 |
|  | GO:0007156 | homophilic cell adhesion | 7.65E‐05 |
| Chr8q23.1‐23.3 | GO:0003160 | endocardium morphogenesis | 0.004996243 |
|  | GO:0003181 | atrioventricular valve morphogenesis | 0.002001375 |
|  | GO:0003192 | mitral valve formation | 0.005992617 |
|  | GO:0003195 | tricuspid valve formation | 0.003998912 |
|  | GO:0003221 | right ventricular cardiac muscle tissue morphogenesis | 0.007982496 |
|  | GO:0006413 | translational initiation | 0.003000623 |
|  | GO:0007171 | activation of transmembrane receptor protein tyrosine kinase activity | 0.002001375 |
|  | GO:0014842 | regulation of satellite cell proliferation | 0.001001168 |
|  | GO:0030210 | heparin biosynthetic process | 0.008976004 |
|  | GO:0035025 | positive regulation of Rho protein signal transduction | 0.003998912 |
|  | GO:0048014 | Tie receptor signaling pathway | 0.005992617 |
|  | GO:0060437 | lung growth | 0.005992617 |
|  | GO:0060535 | trachea cartilage morphogenesis | 0.003000623 |
|  | GO:0060979 | vasculogenesis involved in coronary vascular morphogenesis | 0.003998912 |
|  | GO:0071168 | protein localization to chromatin | 0.004996243 |
|  | GO:0090073 | positive regulation of protein homodimerization activity | 0.003489032 |
|  | GO:2000020 | positive regulation of male gonad development | 0.006988034 |
|  | GO:2000195 | negative regulation of female gonad development | 7.44E‐05 |
|  | GO:2000352 | negative regulation of endothelial cell apoptotic process | 0.003998912 |
| Chr22q12.3‐13.33 | GO:0000460 | maturation of 5.8S rRNA | 0.007627981 |
|  | GO:0001977 | renal system process involved in regulation of blood volume | 0.008700155 |
|  | GO:0055114 | oxidation‐reduction process | 0.008700155 |
|  | GO:0006488 | dolichol‐linked oligosaccharide biosynthetic process | 0.001320887 |
|  | GO:0007127 | meiosis I | 0.002952744 |
|  | GO:0007338 | single fertilization | 0.000268112 |
|  | GO:0010885 | regulation of cholesterol storage | 0.008618932 |
|  | GO:0030520 | intracellular estrogen receptor signaling pathway | 0.001666252 |
|  | GO:0032318 | regulation of Ras GTPase activity | 0.006321168 |
|  | GO:0032342 | aldosterone biosynthetic process | 0.000969853 |
|  | GO:0034651 | cortisol biosynthetic process | 0.008700155 |
|  | GO:0042312 | regulation of vasodilation | 0.005793965 |
|  | GO:0042692 | muscle cell differentiation | 0.007616333 |
|  | GO:0043009 | chordate embryonic development | 0.004545609 |
|  | GO:0044237 | cellular metabolic process | 0.001008483 |
|  | GO:0045017 | glycerolipid biosynthetic process | 0.00990999 |
|  | GO:0045040 | protein import into mitochondrial outer membrane | 0.005793965 |
|  | GO:0055075 | potassium ion homeostasis | 0.001008483 |
|  | GO:0060252 | positive regulation of glial cell proliferation | 0.004670999 |
|  | GO:0071103 | DNA conformation change | 0.003156001 |
|  | GO:0072367 | regulation of lipid transport by regulation of transcription from RNA polymerase II promoter | 0.006606969 |
|  | GO:2000188 | regulation of cholesterol homeostasis | 0.001008483 |
|  | GO:2000311 | regulation of alpha‐amino‐3‐hydroxy‐5‐methyl‐4‐isoxazole propionate selective glutamate receptor activity | 0.005793965 |
| Chr12q13.11‐13.13 | GO:0000915 | cytokinesis, actomyosin contractile ring assembly | 0.005761312 |
|  | GO:0001501 | skeletal system development | 0.005761312 |
|  | GO:0002543 | activation of blood coagulation via clotting cascade | 0.004323183 |
|  | GO:0003430 | growth plate cartilage chondrocyte growth | 0.007675622 |
|  | GO:0016055 | Wnt receptor signaling pathway | 0.001199752 |
|  | GO:0006337 | nucleosome disassembly | 0.002953676 |
|  | GO:0006355 | regulation of transcription, DNA‐dependent | 0.001403458 |
|  | GO:0006814 | sodium ion transport | 0.000231457 |
|  | GO:0006833 | water transport | 0.007675622 |
|  | GO:0007108 | cytokinesis, initiation of separation | 0.000493919 |
|  | GO:0009415 | response to water stimulus | 0.007675622 |
|  | GO:0009952 | anterior/posterior pattern specification | 0.007675622 |
|  | GO:0010980 | positive regulation of vitamin D 24‐hydroxylase activity | 0.007675622 |
|  | GO:0014835 | myoblast cell differentiation involved in skeletal muscle regeneration | 0.004323183 |
|  | GO:0015676 | vanadium ion transport | 0.001348835 |
|  | GO:0015692 | lead ion transport | 0.000577126 |
|  | GO:0015706 | nitrate transport | 0.006551291 |
|  | GO:0021588 | cerebellum formation | 0.007675622 |
|  | GO:0022004 | midbrain‐hindbrain boundary maturation during brain development | 0.008710902 |
|  | GO:0023014 | signal transduction by phosphorylation | 0.009010687 |
|  | GO:0030500 | regulation of bone mineralization | 0.007675622 |
|  | GO:0030858 | positive regulation of epithelial cell differentiation | 0.000348035 |
|  | GO:0031424 | keratinization | 0.007675622 |
|  | GO:0032980 | keratinocyte activation | 0.007675622 |
|  | GO:0034621 | cellular macromolecular complex subunit organization | 0.001591597 |
|  | GO:0034695 | response to prostaglandin E stimulus | 0.007675622 |
|  | GO:0035280 | miRNA loading onto RISC involved in gene silencing by miRNA | 0.002036018 |
|  | GO:0035444 | nickel cation transmembrane transport | 0.007675622 |
|  | GO:0043000 | Golgi to plasma membrane CFTR protein transport | 0.007387534 |
|  | GO:0043401 | steroid hormone mediated signaling pathway | 0.007675622 |
|  | GO:0043534 | blood vessel endothelial cell migration | 0.008281805 |
|  | GO:0043586 | tongue development | 0.009591084 |
|  | GO:0043981 | histone H4‐K5 acetylation | 0.007675622 |
|  | GO:0043982 | histone H4‐K8 acetylation | 0.003610215 |
|  | GO:0043984 | histone H4‐K16 acetylation | 0.007675622 |
|  | GO:0045109 | intermediate filament organization | 0.001199752 |
|  | GO:0046688 | response to copper ion | 0.009152821 |
|  | GO:0045618 | positive regulation of keratinocyte differentiation | 0.001677314 |
|  | GO:0045944 | positive regulation of transcription from RNA polymerase II promoter | 0.007675622 |
|  | GO:0048562 | embryonic organ morphogenesis | 0.004676118 |
|  | GO:0048706 | embryonic skeletal system development | 0.006830403 |
|  | GO:0050687 | negative regulation of defense response to virus | 0.001591597 |
|  | GO:0051885 | positive regulation of anagen | 0.003676575 |
|  | GO:0060558 | regulation of calcidiol 1‐monooxygenase activity | 0.000174902 |

|  | GO:0070564 | positive regulation of vitamin D receptor signaling pathway | 0.007675622 |
| --- | --- | --- | --- |
|  | GO:0070627 | ferrous iron import | 0.007675622 |
|  | GO:0071320 | cellular response to cAMP | 0.007675622 |
|  | GO:0071356 | cellular response to tumor necrosis factor | 0.007648747 |
|  | GO:0071425 | hemopoietic stem cell proliferation | 0.008269291 |
|  | GO:0071773 | cellular response to BMP stimulus | 0.003079314 |
|  | GO:0097045 | phosphatidylserine exposure on blood platelet | 0.007675622 |
|  | GO:1901165 | positive regulation of trophoblast cell migration | 0.007675622 |
|  | GO:2000002 | negative regulation of DNA damage checkpoint | 4.39E‐06 |
| Chr3p12.3‐q13.12 | GO:0000117 | regulation of transcription involved in G2/M‐phase of mitotic cell cycle | 0.004408154 |
|  | GO:0001542 | ovulation from ovarian follicle | 3.38E‐07 |
|  | GO:0007155 | cell adhesion | 0.002861726 |
|  | GO:0007186 | G‐protein coupled receptor signaling pathway | 3.84E‐05 |
|  | GO:0016199 | axon midline choice point recognition | 4.23E‐11 |
|  | GO:0016578 | histone deubiquitination | 0.000114657 |
|  | GO:0035235 | ionotropic glutamate receptor signaling pathway | 0.006215585 |
|  | GO:0043480 | pigment accumulation in tissues | 0.006215585 |
|  | GO:0050911 | detection of chemical stimulus involved in sensory perception of smell | 0.004349187 |
|  | GO:0050925 | negative regulation of negative chemotaxis | 0.005500282 |
|  | GO:0060218 | hemopoietic stem cell differentiation | 0.000150115 |
| Chr21q22.11‐22.3 | GO:0000073 | spindle pole body separation | 0.008361717 |
|  | GO:0000720 | pyrimidine dimer repair by nucleotide‐excision repair | 0.008232874 |
|  | GO:0001508 | regulation of action potential | 0.002794927 |
|  | GO:0009615 | response to virus | 7.70E‐06 |
|  | GO:0016191 | synaptic vesicle uncoating | 0.002794927 |
|  | GO:0014015 | positive regulation of gliogenesis | 0.002794927 |
|  | GO:0015798 | myo‐inositol transport | 0.00938819 |
|  | GO:0021530 | spinal cord oligodendrocyte cell fate specification | 0.005582159 |
|  | GO:0034765 | regulation of ion transmembrane transport | 0.009553413 |
|  | GO:0042376 | phylloquinone catabolic process | 0.002794927 |
|  | GO:0042776 | mitochondrial ATP synthesis coupled proton transport | 0.008361717 |
|  | GO:0042985 | negative regulation of amyloid precursor protein biosynthetic process | 0.005881785 |
|  | GO:0044237 | cellular metabolic process | 0.005316435 |
|  | GO:0045541 | negative regulation of cholesterol biosynthetic process | 0.005582159 |
|  | GO:0048597 | post‐embryonic camera‐type eye morphogenesis | 0.002794927 |
|  | GO:0048842 | positive regulation of axon extension involved in axon guidance | 0.008361717 |
|  | GO:0060047 | heart contraction | 0.005582159 |
|  | GO:0060060 | post‐embryonic retina morphogenesis in camera‐type eye | 0.008361717 |
|  | GO:0070593 | dendrite self‐avoidance | 0.003856439 |
|  | GO:0070781 | response to biotin | 0.008361717 |
|  | GO:0071110 | histone biotinylation | 0.000688259 |
|  | GO:0071336 | regulation of hair follicle cell proliferation | 0.002794927 |
|  | GO:0071435 | potassium ion export | 0.005582159 |
|  | GO:0071805 | potassium ion transmembrane transport | 0.008361717 |
|  | GO:2000872 | positive regulation of progesterone secretion | 0.002794927 |
| Chr5q32 | GO:0000271 | polysaccharide biosynthetic process | 0.00374984 |
|  | GO:0001503 | ossification | 0.001574882 |
|  | GO:0001894 | tissue homeostasis | 0.00125146 |
|  | GO:0002024 | diet induced thermogenesis | 0.009969387 |
|  | GO:0002025 | vasodilation by norepinephrine‐epinephrine involved in regulation of systemic arterial blood pressure | 0.008728488 |
|  | GO:0002032 | desensitization of G‐protein coupled receptor protein signaling pathway by arrestin | 0.00374984 |
|  | GO:0002086 | diaphragm contraction | 0.002501406 |
|  | GO:0002606 | positive regulation of dendritic cell antigen processing and presentation | 0.002501406 |
|  | GO:0002830 | positive regulation of type 2 immune response | 0.007486085 |
|  | GO:0002906 | negative regulation of mature B cell apoptotic process | 0.002501406 |
|  | GO:0003059 | positive regulation of the force of heart contraction by epinephrine | 0.008728488 |
|  | GO:0035811 | negative regulation of urine volume | 0.002501406 |
|  | GO:0030036 | actin cytoskeleton organization | 0.007486085 |
|  | GO:0006390 | transcription from mitochondrial promoter | 0.006242178 |
|  | GO:0006477 | protein sulfation | 0.004996763 |
|  | GO:0006954 | inflammatory response | 0.004996763 |
|  | GO:0007189 | adenylate cyclase‐activating G‐protein coupled receptor signaling pathway | 0.009619541 |
|  | GO:0008284 | positive regulation of cell proliferation | 0.00374984 |
|  | GO:0010694 | positive regulation of alkaline phosphatase activity | 0.002501406 |
|  | GO:0048015 | phosphatidylinositol‐mediated signaling | 0.001526649 |
|  | GO:0030203 | glycosaminoglycan metabolic process | 0.00138621 |
|  | GO:0030328 | prenylcysteine catabolic process | 0.000828468 |
|  | GO:0030593 | neutrophil chemotaxis | 0.00374984 |
|  | GO:0032287 | peripheral nervous system myelin maintenance | 0.004996763 |
|  | GO:0032516 | positive regulation of phosphoprotein phosphatase activity | 0.003765202 |
|  | GO:0035441 | cell migration involved in vasculogenesis | 0.007486085 |
|  | GO:0035524 | proline transmembrane transport | 0.001318632 |
|  | GO:0035691 | macrophage migration inhibitory factor signaling pathway | 0.009477561 |
|  | GO:0035793 | positive regulation of metanephric mesenchymal cell migration by platelet‐derived growth factor receptor‐beta signaling pathway | 0.008114011 |
|  | GO:0036120 | cellular response to platelet‐derived growth factor stimulus | 0.004969346 |
|  | GO:0038091 | positive regulation of cell proliferation by VEGF‐activated platelet derived growth factor receptor signaling pathway | 0.004724861 |
|  | GO:0038145 | macrophage colony‐stimulating factor signaling pathway | 0.00125146 |
|  | GO:0042790 | transcription of nuclear large rRNA transcript from RNA polymerase I promoter | 0.006242178 |
|  | GO:0045087 | innate immune response | 0.007631182 |
|  | GO:0045124 | regulation of bone resorption | 0.008728488 |
|  | GO:0045217 | cell‐cell junction maintenance | 0.00125146 |
|  | GO:0045292 | nuclear mRNA cis splicing, via spliceosome | 0.001716558 |
|  | GO:0045672 | positive regulation of osteoclast differentiation | 9.07E‐06 |
|  | GO:0046037 | GMP metabolic process | 0.006299998 |
|  | GO:0046488 | phosphatidylinositol metabolic process | 0.00777192 |
|  | GO:0046777 | protein autophosphorylation | 0.009969387 |
|  | GO:0048633 | positive regulation of skeletal muscle tissue growth | 0.004502911 |
|  | GO:0048660 | regulation of smooth muscle cell proliferation | 0.009969387 |
|  | GO:0048702 | embryonic neurocranium morphogenesis | 0.000162995 |
|  | GO:0048705 | skeletal system morphogenesis | 0.009969387 |
|  | GO:0050730 | regulation of peptidyl‐tyrosine phosphorylation | 0.008728488 |

|  | GO:0050921 | positive regulation of chemotaxis | 0.006242178 |
| --- | --- | --- | --- |
|  | GO:0051254 | positive regulation of RNA metabolic process | 0.000482946 |
|  | GO:0060041 | retina development in camera‐type eye | 0.004393743 |
|  | GO:0060414 | aorta smooth muscle tissue morphogenesis | 0.00125146 |
|  | GO:0060907 | positive regulation of macrophage cytokine production | 0.00374984 |
|  | GO:0060947 | cardiac vascular smooth muscle cell differentiation | 0.007486085 |
|  | GO:0060981 | cell migration involved in coronary angiogenesis | 6.38E‐07 |
|  | GO:0070374 | positive regulation of ERK1 and ERK2 cascade | 0.006242178 |
|  | GO:0071391 | cellular response to estrogen stimulus | 0.007486085 |
|  | GO:0071670 | smooth muscle cell chemotaxis | 0.008728488 |
|  | GO:0071902 | positive regulation of protein serine/threonine kinase activity | 0.001094908 |
|  | GO:0072262 | metanephric glomerular mesangial cell proliferation involved in metanephros development | 0.002908607 |
|  | GO:0072277 | metanephric glomerular capillary formation | 0.000255491 |
|  | GO:0090197 | positive regulation of chemokine secretion | 0.004433048 |
|  | GO:0090280 | positive regulation of calcium ion import | 0.000212705 |
|  | GO:1900017 | positive regulation of cytokine production involved in inflammatory response | 0.004996763 |
|  | GO:2000249 | regulation of actin cytoskeleton reorganization | 0.008728488 |
|  | GO:2000343 | positive regulation of chemokine (C‐X‐C motif) ligand 2 production | 0.006242178 |
|  | GO:2000573 | positive regulation of DNA biosynthetic process | 0.00374984 |
| Chr18q21.2 | GO:0000122 | negative regulation of transcription from RNA polymerase II promoter | 0.004122933 |
|  | GO:0006139 | nucleobase‐containing compound metabolic process | 0.004496995 |
|  | GO:0010740 | positive regulation of intracellular protein kinase cascade | 0.000750751 |
|  | GO:0010909 | positive regulation of heparan sulfate proteoglycan biosynthetic process | 0.007484995 |
|  | GO:0014003 | oligodendrocyte development | 0.001875937 |
|  | GO:0021965 | spinal cord ventral commissure morphogenesis | 0.007190211 |
|  | GO:0030111 | regulation of Wnt receptor signaling pathway | 0.004496995 |
|  | GO:0030538 | embryonic genitalia morphogenesis | 0.009067483 |
|  | GO:0031641 | regulation of myelination | 0.009720762 |
|  | GO:0032252 | secretory granule localization | 0.008230749 |
|  | GO:0033563 | dorsal/ventral axon guidance | 0.000750751 |
|  | GO:0035411 | catenin import into nucleus | 0.000750751 |
|  | GO:0038007 | netrin‐activated signaling pathway | 0.001125938 |
|  | GO:0040037 | negative regulation of fibroblast growth factor receptor signaling pathway | 0.00084904 |
|  | GO:0042711 | maternal behavior | 0.001741941 |
|  | GO:0043570 | maintenance of DNA repeat elements | 0.007672989 |
|  | GO:0044334 | canonical Wnt receptor signaling pathway involved in positive regulation of epithelial to mesenchymal transition | 0.001501 |
|  | GO:0045664 | regulation of neuron differentiation | 0.001501 |
|  | GO:0046621 | negative regulation of organ growth | 0.003347227 |
|  | GO:0046827 | positive regulation of protein export from nucleus | 0.001501 |
|  | GO:0048557 | embryonic digestive tract morphogenesis | 0.001501 |
|  | GO:0048619 | embryonic hindgut morphogenesis | 0.003374434 |
|  | GO:0048625 | myoblast cell fate commitment | 0.001501 |
|  | GO:0051047 | positive regulation of secretion | 0.008230749 |
|  | GO:0071985 | multivesicular body sorting pathway | 0.001501 |
| Chr18q22.2‐22.3 | GO:0002860 | positive regulation of natural killer cell mediated cytotoxicity directed against tumor cell target | 0.003499268 |
|  | GO:0002891 | positive regulation of immunoglobulin mediated immune response | 0.005244962 |
|  | GO:0006662 | glycerol ether metabolic process | 0.005826277 |
|  | GO:0033005 | positive regulation of mast cell activation | 0.009308058 |
|  | GO:0048169 | regulation of long‐term neuronal synaptic plasticity | 0.001750948 |
|  | GO:0051965 | positive regulation of synapse assembly | 0.006407301 |
|  | GO:0060369 | positive regulation of Fc receptor mediated stimulatory signaling pathway | 0.000583942 |
| Chr9q33.3‐34.3 | GO:0001516 | prostaglandin biosynthetic process | 0.002501422 |
|  | GO:0006405 | RNA export from nucleus | 0.008890307 |
|  | GO:0006957 | complement activation, alternative pathway | 0.002501422 |
|  | GO:0009133 | nucleoside diphosphate biosynthetic process | 0.008890307 |
|  | GO:0009179 | purine ribonucleoside diphosphate metabolic process | 3.72E‐06 |
|  | GO:0009191 | ribonucleoside diphosphate catabolic process | 0.004100568 |
|  | GO:0010700 | negative regulation of norepinephrine secretion | 0.000730687 |
|  | GO:0010832 | negative regulation of myotube differentiation | 0.00752609 |
|  | GO:0042981 | regulation of apoptotic process | 0.003310759 |
|  | GO:0019348 | dolichol metabolic process | 0.005753992 |
|  | GO:0032495 | response to muramyl dipeptide | 0.008561546 |
|  | GO:0035305 | negative regulation of dephosphorylation | 0.001208851 |
|  | GO:0044237 | cellular metabolic process | 0.001208851 |
|  | GO:0050794 | regulation of cellular process | 1.38E‐05 |
|  | GO:0051085 | chaperone mediated protein folding requiring cofactor | 0.006335658 |
|  | GO:0051179 | localization | 0.004225486 |
|  | GO:0060528 | secretory columnal luminar epithelial cell differentiation involved in prostate glandular acinus development | 0.006120555 |
|  | GO:0070085 | glycosylation | 0.001799949 |
|  | GO:0071763 | nuclear membrane organization | 0.006740372 |
|  | GO:0090101 | negative regulation of transmembrane receptor protein serine/threonine kinase signaling pathway | 0.007577914 |
|  | GO:1900116 | extracellular negative regulation of signal transduction | 0.004225486 |
| Chr16q21‐24.3;  Chr1q42.13‐42.2 | GO:0002040 | sprouting angiogenesis | 0.001449827 |
|  | GO:0002198 | S/G2 transition of mitotic cell cycle | 0.00899737 |
|  | GO:0002316 | follicular B cell differentiation | 0.008687325 |
|  | GO:0003106 | negative regulation of glomerular filtration by angiotensin | 0.008885366 |
|  | GO:0003331 | positive regulation of extracellular matrix constituent secretion | 0.008885366 |
|  | GO:0003356 | regulation of cilium beat frequency | 0.000429014 |
|  | GO:0006084 | acetyl‐CoA metabolic process | 0.006571994 |
|  | GO:0006430 | lysyl‐tRNA aminoacylation | 0.008885366 |
|  | GO:0006546 | glycine catabolic process | 0.008885366 |
|  | GO:0006801 | superoxide metabolic process | 0.00172853 |
|  | GO:0006891 | intra‐Golgi vesicle‐mediated transport | 0.008885366 |
|  | GO:0007057 | spindle assembly involved in female meiosis I | 0.005364823 |
|  | GO:0007160 | cell‐matrix adhesion | 0.008885366 |
|  | GO:0007202 | activation of phospholipase C activity | 0.008885366 |
|  | GO:0007494 | midgut development | 0.007645863 |
|  | GO:0010811 | positive regulation of cell‐substrate adhesion | 0.008885366 |
|  | GO:0014822 | detection of wounding | 0.008885366 |

|  | GO:0014873 | response to muscle activity involved in regulation of muscle adaptation | 0.002883208 |
| --- | --- | --- | --- |
|  | GO:0014895 | smooth muscle hypertrophy | 0.008885366 |
|  | GO:0015031 | protein transport | 0.002124093 |
|  | GO:0015938 | coenzyme A catabolic process | 0.008885366 |
|  | GO:0016999 | antibiotic metabolic process | 0.008885366 |
|  | GO:0018146 | keratan sulfate biosynthetic process | 0.008885366 |
|  | GO:0021852 | pyramidal neuron migration | 0.004836225 |
|  | GO:0030163 | protein catabolic process | 0.002715018 |
|  | GO:0030851 | granulocyte differentiation | 0.008885366 |
|  | GO:0031570 | DNA integrity checkpoint | 0.008885366 |
|  | GO:0031848 | protection from non‐homologous end joining at telomere | 0.008885366 |
|  | GO:0032205 | negative regulation of telomere maintenance | 0.008885366 |
|  | GO:0034470 | ncRNA processing | 0.008885366 |
|  | GO:0035729 | cellular response to hepatocyte growth factor stimulus | 0.008885366 |
|  | GO:0036158 | outer dynein arm assembly | 0.002715018 |
|  | GO:0036159 | inner dynein arm assembly | 0.008885366 |
|  | GO:0042384 | cilium assembly | 0.008885366 |
|  | GO:0043542 | endothelial cell migration | 0.008885366 |
|  | GO:0044249 | cellular biosynthetic process | 0.008885366 |
|  | GO:0045403 | negative regulation of interleukin‐4 biosynthetic process | 0.008885366 |
|  | GO:0046112 | nucleobase biosynthetic process | 0.008885366 |
|  | GO:0048010 | vascular endothelial growth factor receptor signaling pathway | 0.001151337 |
|  | GO:0048012 | hepatocyte growth factor receptor signaling pathway | 0.007645863 |
|  | GO:0048213 | Golgi vesicle prefusion complex stabilization | 0.008885366 |
|  | GO:0048613 | embryonic ectodermal digestive tract morphogenesis | 0.00077208 |
|  | GO:0048738 | cardiac muscle tissue development | 0.006573939 |
|  | GO:0051442 | negative regulation of ubiquitin‐protein ligase activity involved in meiotic cell cycle | 0.001151337 |
|  | GO:0060377 | negative regulation of mast cell differentiation | 0.008885366 |
|  | GO:0060438 | trachea development | 0.00456749 |
|  | GO:0060446 | branching involved in open tracheal system development | 0.002190558 |
|  | GO:0060461 | right lung morphogenesis | 0.004291511 |
|  | GO:0060565 | inhibition of mitotic anaphase‐promoting complex activity | 0.001151337 |
|  | GO:0070198 | protein localization to chromosome, telomeric region | 0.004383379 |
|  | GO:0070830 | tight junction assembly | 0.005364823 |
|  | GO:0072011 | glomerular endothelium development | 0.00077208 |
|  | GO:0072512 | trivalent inorganic cation transport | 0.005059872 |
|  | GO:0090131 | mesenchyme migration | 0.008885366 |
|  | GO:0090162 | establishment of epithelial cell polarity | 0.005746483 |
|  | GO:0090304 | nucleic acid metabolic process | 0.008099472 |
|  | GO:0090410 | malonate catabolic process | 0.005750918 |
|  | GO:2000147 | positive regulation of cell motility | 0.009918539 |
|  | GO:2000296 | negative regulation of hydrogen peroxide catabolic process | 0.008885366 |
|  | GO:2000602 | regulation of interphase of mitotic cell cycle | 0.003165288 |
| Chr11q23.1-24.1 | GO:0000077 | DNA damage checkpoint | 0.000176809 |
|  | GO:0000083 | regulation of transcription involved in G1/S phase of mitotic cell cycle | 0.001212695 |
|  | GO:0007157 | heterophilic cell-cell adhesion | 0.001212695 |
|  | GO:0001666 | response to hypoxia | 0.00662032 |
|  | GO:0002355 | detection of tumor cell | 0.007176514 |
|  | GO:0005991 | trehalose metabolic process | 0.005822177 |
|  | GO:0006084 | acetyl-CoA metabolic process | 0.007717337 |
|  | GO:0006650 | glycerophospholipid metabolic process | 0.007717337 |
|  | GO:0006783 | heme biosynthetic process | 0.00256381 |
|  | GO:0006810 | transport | 0.004448459 |
|  | GO:0007210 | serotonin receptor signaling pathway | 0.003192947 |
|  | GO:0008203 | cholesterol metabolic process | 2.93E-05 |
|  | GO:0008624 | induction of apoptosis by extracellular signals | 0.000870631 |
|  | GO:0009306 | protein secretion | 0.007717337 |
|  | GO:0010873 | positive regulation of cholesterol esterification | 0.007717337 |
|  | GO:0010898 | positive regulation of triglyceride catabolic process | 0.002897123 |
|  | GO:0010902 | positive regulation of very-low-density lipoprotein particle remodeling | 0.007717337 |
|  | GO:0010903 | negative regulation of very-low-density lipoprotein particle remodeling | 0.007717337 |
|  | GO:0010987 | negative regulation of high-density lipoprotein particle clearance | 0.007717337 |
|  | GO:0014076 | response to fluoxetine | 0.007142575 |
|  | GO:0016116 | carotenoid metabolic process | 0.007717337 |
|  | GO:0016121 | carotene catabolic process | 0.000870631 |
|  | GO:0016338 | calcium-independent cell-cell adhesion | 7.13E-05 |
|  | GO:0018160 | peptidyl-pyrromethane cofactor linkage | 0.007717337 |
|  | GO:0030300 | regulation of intestinal cholesterol absorption | 0.002057868 |
|  | GO:0031283 | negative regulation of guanylate cyclase activity | 0.007465169 |
|  | GO:0032488 | Cdc42 protein signal transduction | 0.000400061 |
|  | GO:0033344 | cholesterol efflux | 0.001212695 |
|  | GO:0033490 | cholesterol biosynthetic process via lathosterol | 0.002230007 |
|  | GO:0033700 | phospholipid efflux | 0.003112189 |
|  | GO:0035095 | behavioral response to nicotine | 0.000870631 |
|  | GO:0035725 | sodium ion transmembrane transport | 0.007717337 |
|  | GO:0042157 | lipoprotein metabolic process | 2.46E-05 |
|  | GO:0042632 | cholesterol homeostasis | 0.007717337 |
|  | GO:0043691 | reverse cholesterol transport | 0.000176809 |
|  | GO:0045723 | positive regulation of fatty acid biosynthetic process | 5.24E-05 |
|  | GO:0046641 | positive regulation of alpha-beta T cell proliferation | 0.001351023 |
|  | GO:0048513 | organ development | 3.67E-05 |
|  | GO:0050713 | negative regulation of interleukin-1 beta secretion | 0.003715723 |
|  | GO:0051006 | positive regulation of lipoprotein lipase activity | 0.002057868 |
|  | GO:0051569 | regulation of histone H3-K4 methylation | 0.003715723 |
|  | GO:0060084 | synaptic transmission involved in micturition | 0.007717337 |
|  | GO:0060192 | negative regulation of lipase activity | 0.000583385 |
|  | GO:0060354 | negative regulation of cell adhesion molecule production | 0.005822177 |
|  | GO:0060371 | regulation of atrial cardiomyocyte membrane depolarization | 3.67E-05 |
|  | GO:0065005 | protein-lipid complex assembly | 3.67E-05 |
|  | GO:0070328 | triglyceride homeostasis | 0.007717337 |
|  | GO:0070508 | cholesterol import | 0.006930571 |
|  | GO:2000377 | regulation of reactive oxygen species metabolic process | 0.005071509 |
|  | GO:0080182 | histone H3-K4 trimethylation | 0.001763694 |

|  | GO:0086002 | regulation of cardiac muscle cell action potential involved in contraction | 0.006444365 |
| --- | --- | --- | --- |
|  | GO:0086065 | cell communication involved in cardiac conduction | 5.02E-05 |
|  | GO:0086091 | regulation of heart rate by cardiac conduction | 0.002269307 |
|  | GO:0090072 | positive regulation of sodium ion transport via voltage-gated sodium channel activity | 0.000122176 |
|  | GO:2000256 | positive regulation of male germ cell proliferation | 0.007717337 |
|  | GO:2000649 | regulation of sodium ion transmembrane transporter activity | 0.000870631 |
|  | GO:2001040 | positive regulation of cellular response to drug | 0.008355963 |
